# A conserved mechanism determines the activity of two pivotal transcription factors that control epidermal cell differentiation in *Arabidopsis thaliana*

**DOI:** 10.1007/s10265-023-01439-7

**Published:** 2023-02-24

**Authors:** Kenji Nagata, Mitsutomo Abe

**Affiliations:** Department of Life Sciences, Graduate School of Arts and Sciences, The University of Tokyo, 3-8-1, Komaba, Meguro-Ku, Tokyo, 153-8902 Japan

**Keywords:** *Arabidopsis thaliana*, Epidermis, PROTODERMAL FACTOR2 (PDF2), Steroidogenic acute regulatory protein-related lipid transfer (START) domain

## Abstract

The surface of plants is covered by the epidermis, which protects the plant’s body from the external environment and mediates inter-cell layer signaling to regulate plant development. Therefore, the manifestation of epidermal traits at a precise location is a prerequisite for their normal growth and development. In *Arabidopsis thaliana*, class IV homeodomain-leucine zipper transcription factors PROTODERMAL FACTOR2 (PDF2) and ARABIDOPSIS THALIANA MERISTEM LAYER1 (ATML1) play redundant roles in epidermal cell differentiation. Nevertheless, several pieces of evidence suggest that the activity and/or function of PDF2 and ATML1 are regulated differently. The role of the steroidogenic acute regulatory protein-related lipid transfer (START) domain of ATML1 in restricting this protein’s activity has been demonstrated; however, whether this lipid-dependent mechanism regulates PDF2 expression is unknown. In this study, we demonstrated that the START domains of PDF2 and ATML1, regulate protein turnover in a position-dependent manner and affect the dimeric proteins. Our results show that a conserved mechanism provides the basis for the functional redundancy of PDF2 and ATML1 in epidermal cell differentiation and that an unidentified regulatory layer specific to PDF2 or ATML1 is responsible for the difference in the activity and/or function of PDF2 and ATML1.

**Supplementary Information:**

The online version contains supplementary material available at 10.1007/s10265-023-01439-7.

## Introduction

Terrestrial plants can be found in harsh environments. Therefore, to protect them from hostile conditions, the surface of plants is covered with an epidermis, generally composed of a single layer of epidermal cells that express a variety of defensive traits. Epidermal cells deposit a cuticle, which is made up of cutin polyester and wax on the outer cell walls (Javelle et al. 2011). Lipophilic cuticles act as diffusion barriers to prevent water loss from the plant’s body to dry environments and are the first line of defense against harmful invaders, such as pathogens and pests (Yeats and Rose 2013). Thus, the differentiation of defensive epidermal cells at the boundary between the environment and the plant body is crucial for the survival of terrestrial plants.

Moreover, the epidermis not only acts as a defensive cell layer but also serves as a critical signaling hub for regulating multiple aspects of plant development. For example, several mutants of the angiosperm *Arabidopsis thaliana* (Arabidopsis) with impaired epidermal cell differentiation result in lethality due to the disordered structure and disrupted function of the shoot apical meristem (SAM) (Abe et al. 2003; Ogawa et al. 2015). Recently, it was reported that epidermis-derived inter-cell layer signaling is required to maintain the structure and function of SAM (Han et al. 2020); thus, an intact epidermis is necessary not only for the execution of its function but also for the normal development of plants.

In Arabidopsis, the differentiation of epidermal cells is regulated by a pair of class IV homeodomain-leucine zipper (HD-ZIP IV) transcription factors (TFs): PROTODERMAL FACTOR 2 (PDF2) and ARABIDOPSIS THALIANA MERISTEM LAYER1 (ATML1) (Abe et al. 2001, 2003; Nagata et al. 2021; Ogawa et al. 2015; Takada et al. 2013). PDF2 and ATML1 are expressed predominantly in the outermost cell layer of aerial organs (Abe et al. 2003; Lu et al. 1996; Nagata et al. 2021; Sessions et al. 1999) and immature roots before root cap sloughing (Nagata et al. 2021). These TFs orchestrate the expression of epidermal cell-specific genes, some of which are involved in cuticle formation (e.g., *FIDDLEHEAD*, *3-KETOACYL-COA SYNTHASE 20, PASTICCINO 2*) (Nagata and Abe 2021; Nagata et al. 2021; Rombolá-Caldentey et al. 2014), as well as maintaining SAM structure and function (e.g., *microRNA171* family genes) (Han et al. 2020). Consistently, hypomorphic *pdf2-1; atml1-1* double mutants lack an epidermal cell layer in the aerial tissue (Abe et al. 2003) and show a severe developmental delay of lateral root primordia formation (Nagata et al. 2021). Furthermore, double mutants of a strong *atml1‐3* allele with null *PDF2* alleles result in embryonic lethality (Ogawa et al. 2015). As single mutants of either *PDF2* or *ATML1* do not exhibit such phenotypes, *PDF2* and *ATML1* act redundantly in the differentiation of epidermal cells and are required for normal plant development.

PDF2 and ATML1 contain a homeodomain (HD), a leucine zipper-loop-zipper (ZIP) motif, and a steroidogenic acute regulatory protein-related lipid transfer (START) domain (Ariel et al. 2007; Schrick et al. 2004). The HDs of PDF2 and ATML1 act as DNA-binding domains and specifically bind to the L1 box, a well-conserved *cis*-regulatory element within the promoter regions of epidermal cell-specific genes (Abe et al. 2001, 2003; Han et al. 2020; Nakamura et al. 2006). The ZIP motifs of PDF2 and ATML1, which are located immediately downstream of their HDs, act as dimerization motifs and mediate the homodimerization of PDF2 and ATML1, and heterodimerization between PDF2 and ATML1 (Nagata and Abe 2021; Rombolá-Caldentey et al. 2014; San-Bento et al. 2014). Additionally, the START domains of PDF2 and ATML1 are also lipid-binding and interact with specific lipid ligands (Nagata et al. 2021; Wojciechowska et al. 2021), therefore demonstrating the similarities that each PDF2 and ATML1 domain share in terms of their biochemical properties.

In the past decade, significant progress has been made in understanding the functional significance of the biochemical properties of HD-ZIP IV START domains. First, the START domains of PDF2, ATML1, and GLABRA2 (GL2) bind to lipid ligands to stimulate TF activity in yeast cells (Schrick et al. 2014). Second, the START domains of ATML1 and GL2 bind to their lipid ligands to protect them from turnover and affect the level of dimeric proteins in plant cells (Mukherjee et al. 2022; Nagata and Abe 2021; Nagata et al. 2021). In particular, very-long-chain fatty acid-containing ceramides (VLCFA-Cers), which are lipid ligands in the START domain of PDF2 and ATML1, are thought to be generated predominantly in the outermost cells (Nagata et al. 2021). Consequently, the interaction of the START domain with VLCFA-Cer protects ATML1 from turnover in the outermost cells and increases the level of dimeric ATML1 in the epidermis. Thus, through the lipid-binding properties of the START domain, ATML1 plays a key role in the differentiation of epidermal cells at a precise location.

Although PDF2 and ATML1 share similar biochemical properties and act redundantly in epidermal cell differentiation, it is controversial whether the interaction of the START domain with its specific lipid ligand also induces an alteration of PDF2 turnover and dimerization. Indeed, several studies have suggested that PDF2 has specific functions in epidermal cell differentiation that are distinct from those of ATML1. For example, the overexpression of ATML1 activates the expression of epidermal cell-specific genes, including *ATML1* and *PDF2*, and ectopically induces epidermal cell-related traits in non-epidermal tissues (Peterson et al. 2013; Takada et al. 2013), whereas the overexpression of PDF2 causes a severe delay in flowering time (Abe et al. 2003). Furthermore, double mutants of *PDF2*, in combination with certain *HD-ZIP IV* genes, exhibit defects in stamen development and the specification of petal and stamen identities. However, double mutants of *ATML1*, in combination with *HD-ZIP IV* genes, do not show such defects (Kamata et al. 2013a, b).

Here, we showed that the START domain of PDF2, as well as that of ATML1, regulates protein turnover in a position-dependent manner and affects the dimeric proteins. Thus, a conserved mechanism regulates the activities of PDF2 and ATML1 during epidermal cell differentiation, in which these TFs play redundant roles. Therefore, our results strongly suggest the existence of an unidentified regulatory layer responsible for the functional differences between PDF2 and ATML1.

## Materials and methods

### Plant materials and growth conditions

All transgenic lines used in this study were of the Col-0 background. Transgenic lines for *gPDF2-EGFP; atml1-3; pdf2-1* (#3) were previously described (Nagata et al. 2021). Transgenic lines for *HSP::NLS-mCherry; HSP::PDF2-EGFP* (#1) and *HSP::NLS-mCherry; HSP::PDF2*^*W463L*^*-EGFP* (#5) were obtained in this study. Plants were grown on soil or Murashige and Skoog (MS) solid medium supplemented with 1% sucrose at 23 °C under long-day conditions (16 h light/8 h dark).

### Drug treatment

Cafenstrole (FUJIFILM Wako Pure Chemical Corporation) was dissolved in dimethyl sulfoxide (DMSO) to a concentration of 3 mM and added to the medium. Seedlings were germinated directly on MS solid media supplemented with 0.3 μM cafenstrole. Mock treatment (0.01% [v/v] DMSO treatment) was used as a control.

### Plasmid constructions

To generate the PDF2^W463L^ fragment, two truncated coding sequence (CDS) fragments of PDF2 with the W463L mutation (PDF2-m1 and PDF2-m2) were amplified using PCR. The PDF2-m1 fragment was amplified using the primer sets PDF2-m1-F and PDF2-m1-R (Table S1) and the PDF2-m2 fragment was amplified using the primer sets PDF2-m2-F and PDF2-m2-R (Table S1). The resulting PCR products were cloned into the NdeI/SalI-digested pRI201AN vector (TaKaRa) using GeneArt™ Gibson Assembly HiFi Master Mix (Thermo Fisher Scientific), and the resulting plasmid was used as the PCR template for plasmid construction.

To construct HSP::NLS-mCherry; HSP::PDF2-EGFP or HSP::NLS-mCherry; HSP::PDF2^W463L^-EGFP, the ATML CDS fragment of the vector containing the HSP::NLS-mCherry; HSP::ATML1^WT^-EGFP construct (Nagata et al. 2021) was replaced with the CDS fragment of PDF2 or PDF2^W463L^, respectively.

For the yeast two-hybrid assay, the CDS fragments of ATML1, ATML1^W471L^, PDF2, and PDF2^W463L^ were cloned into the EcoRI and SalI sites of pAD-GAL4-2.1 or pBD-GAL4 Cam (Agilent Technologies) using the GeneArt™ Gibson Assembly HiFi Master Mix.

For the BiFC assay, the CDS fragments of ATML1, ATML1^W471L^, PDF2, and PDF2^W463L^ were cloned into the SpeI sites of p35S::EYFPN or p35S::EYEPC (Nagata et al. 2021) using the GeneArt™ Gibson Assembly HiFi Master Mix.

For the transient overexpression assay, the CDS fragments of ATML1-EGFP, ATML1^W471L^-EGFP, PDF2-EGFP or PDF2^W463L^-EGFP were amplified by PCR using HSP::NLS-mCherry; HSP::ATML1^WT^-EGFP (Nagata et al. 2021), HSP::NLS-mCherry; HSP::ATML1^W471L^-EGFP (Nagata et al. 2021), HSP::NLS-mCherry; HSP::PDF2-EGFP and HSP::NLS-mCherry; HSP::PDF2^W463L^-EGFP as templates, respectively. The resulting PCR products were cloned into the NdeI/SalI-digested pRI201AN vector (TaKaRa) using the GeneArt™ Gibson Assembly HiFi Master Mix.

### Transformation of Arabidopsis

The constructs described above were introduced into *Agrobacterium tumefaciens* strain GV3101 and transformed into Arabidopsis plants using the floral-dip procedure (Clough and Bent 1998).

### Microscopic analysis

Confocal microscopic analysis was performed using a C2 Confocal Microscope (Nikon). Excitation for EGFP and EYFP are 488 nm and mCherry is 561 nm. Emission signals were collected at 500–550 nm for EGFP and EYFP and 575–615 nm for mCherry. Images were processed with NIS-elements (Nikon) and ImageJ software.

### Heat-pulse induction assay

Heat-pulse induction assay was performed as previously described (Nagata et al. 2021). For heat-pulse treatment, 10-day-old seedlings were submerged in 1/2 MS liquid medium and incubated in a water bath for 2 h at 35 °C. Images were obtained 3 h after heat-pulse treatment. Signal intensities of EGFP and mCherry along a line across the nucleus were measured using the [Plot Profile] tools of ImageJ software. All experiments were repeated at least twice with similar results.

### RT-PCR analysis

Total RNA was extracted using TRIzol reagent (Thermo Fisher Scientific). Reverse transcription reactions were carried out using ReverTra Ace qPCR RT Master Mix with a gDNA Remover kit (TOYOBO) according to the manufacturer’s protocol. Primer sequences and PCR conditions are listed in Table S2. All experiments were repeated at least twice with similar results.

### Yeast two-hybrid analysis

The yeast two-hybrid analysis was performed as previously described (Nagata and Abe 2021). Briefly, the appropriate plasmids were transformed into the yeast strain Y2HGold (Takara) using the lithium acetate method. Transformed yeast cells were resolved in 1 × TE buffer, and the cell density was adjusted to an OD at 600 nm of 0.5. The cell suspensions are then diluted 50 times and 5 μl of the dilutions were spotted on SD medium without Leu and Trp or without His, Ala, Leu, and Trp. These plates were incubated at 30 °C for 2 or 4 days. All experiments were repeated twice with similar results.

### Bimolecular fluorescence complementation (BiFC) assay

The BiFC assay was performed as previously described (Nagata and Abe 2021). The appropriate plasmids transformed into *Agrobacterium tumefaciens* strain GV3101, and the resulting strains were used for agroinfiltration of *Nicotiana benthamiana* leaves. Images were obtained on day 2 after infiltration. The empty plasmid (pRI201AN vector (TaKaRa)) was used as a negative control. All microscopic images were acquired using identical settings. The mean signal intensity of EYFP fluorescence in the nuclei was measured using ImageJ software.

### Western blot analysis

To compare the level of ATML1, ATML1^W471L^, PDF2 or PDF2^W463L^ protein expressed under the constitutive CaMV promoter in the *Nicotiana benthamiana* leaves (i.e., transient overexpression assay), the appropriate plasmids were introduced into *Agrobacterium tumefaciens* strain GV3101, and the resulting strains were used for agroinfiltration of *Nicotiana benthamiana* leaves. The empty plasmid (pRI201AN vector (TaKaRa)) was used as a negative control. Samples were collected on day 2 after infiltration, flash frozen in liquid nitrogen, and stored at − 80 °C prior to protein extraction. Protein extractions were performed as previously described (Mukherjee et al. 2022). Briefly, frozen samples were homogenized in liquid nitrogen, and hot sodium dodecyl sulfate (SDS) buffer (8 M urea, 2% SDS, 0.1 M DTT, 20% glycerol, 0.1 M Tris–HCl pH 6.8, 0.004% bromophenol blue, and 5 × Protease Inhibitor Cocktail for General Use (Nacalai tesque)) was added prior to SDS–polyacrylamide gel electrophoresis (PAGE) and Western blotting. Proteins were detected via anti-GFP primary antibody [MBL (598MS); 1:2000] and anti-rabbit IgG HRP-conjugate secondary antibody [Promega (W4018); 1:5000]. Detection of secondary antibodies were performed with the Ez West Lumi One (ATTO) using the Image Quant LAS 4000 mini (GE Healthcare).

## Results

**Fig. 1:**
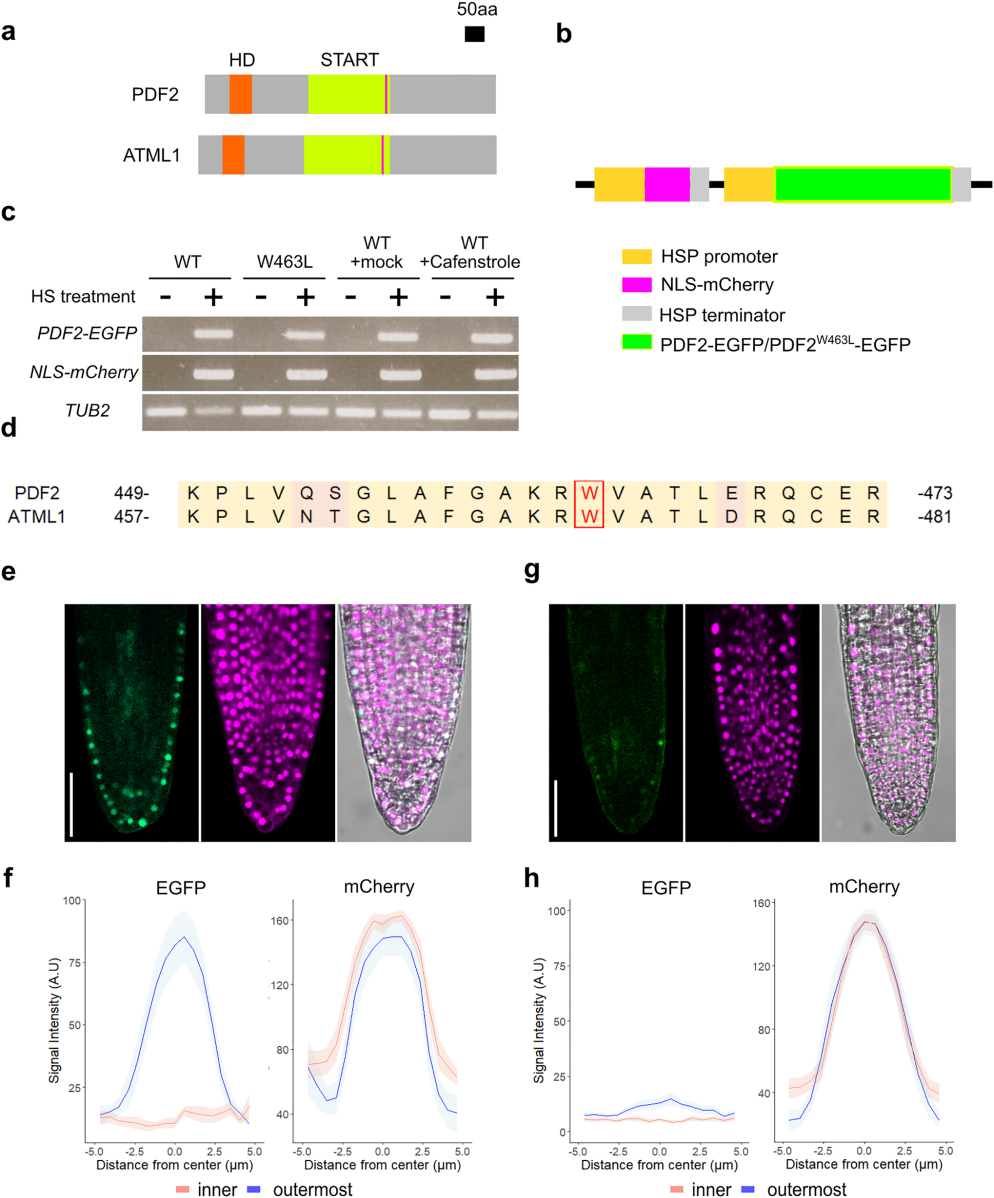
The lipid-binding START domain regulates the turnover of PDF2 protein in a cell position-dependent manner. **a** Representative images of PDF2 (top) and ATML1 (bottom) protein structure. HD, homeodomain; START, START domain. Magenta line indicates the mutation site. **b** Schematic representation of the construct used to visualize the post-translational behavior of NLS-mCherry, PDF2-EGFP and PDF2^W463L^-EGFP by a heat-inducible transient expression system. **c** *PDF2-EGFP* and *NLS-EGFP* expression pre- (−) and post- ( +) the heat-pulse treatment in *HSP::NLS-mCherry; HSP::PDF2-EGFP* plants (WT) or *HSP::NLS-mCherry; HSP::PDF2*^*W463L*^*-EGFP* plants (W463L). *TUB2* (AT5G62690) was amplified for reference. Seedlings were grown on MS medium. To evaluate the effect of cafenstrole treatment on the activity of the *HSP* promoter, seedlings were grown in an MS medium without (+ mock) or with 0.3 µM cafenstrole (+ Cafenstrole). **d** Alignment of the amino acid sequence of the fourth alpha-helix of the START domain from PDF2 and ATML1. Red letter with a red box indicates a tryptophan residue, which is highly conserved among HD-ZIP IV TFs. The yellow background indicates amino acids conserved between PDF2 and ATML1. The pink background color indicates amino acids which are not conserved in PDF2 and ATML1. **e, g** Fluorescent images of EGFP (left) and mCherry (middle), and merged fluorescence and bright-field image (right) in LRs of the transgenic plants 3 h post heat-pulse treatment. **f, h** Plots of EGFP (left) and mCherry (right) intensity in inner cells (*n* = 12) and outermost cells (*n* = 12). *HSP::NLS-mCherry; HSP::PDF2-EGFP* plants are shown in (**e**) and (**f**); *HSP::NLS-mCherry; HSP::PDF2*^*W463L*^*-EGFP* plants are shown in (**g**) and (**h**). Scale bars: 50 μm. Mean data shown (mean ± s.e.m)

To evaluate the turnover of PDF2 protein *in planta*, we first developed a heat-inducible transient expression system in lateral roots (LRs) using the *HEAT SHOCK PROTEIN 18.2* (*HSP*) promoter (Nagata et al. 2021; Takahashi et al. 1992) to induce the enhanced green fluorescent protein (EGFP) fused to PDF2 (PDF2-EGFP) and mCherry with nuclear-localization signal (NLS-mCherry) (*HSP::NLS-mCherry; HSP::PDF2-EGFP*) (Fig. 1a, b). Reverse transcription PCR (RT-PCR) analysis confirmed that the transcription of *PDF2-EGFP* and *NLS-mCherry* were induced in a heat-pulse treatment-dependent manner (Fig. 1c). At 3 h post heat-pulse treatment, EGFP fluorescence was detected predominantly in the outermost cells of the LR. However, mCherry fluorescence, as a positive control, was still detected in all LR cells (Fig. 1e, f). These observations indicate that the turnover of the PDF2 protein is regulated in a cell position-dependent manner, similar to that of the ATML1 protein.

**Fig. 2:**
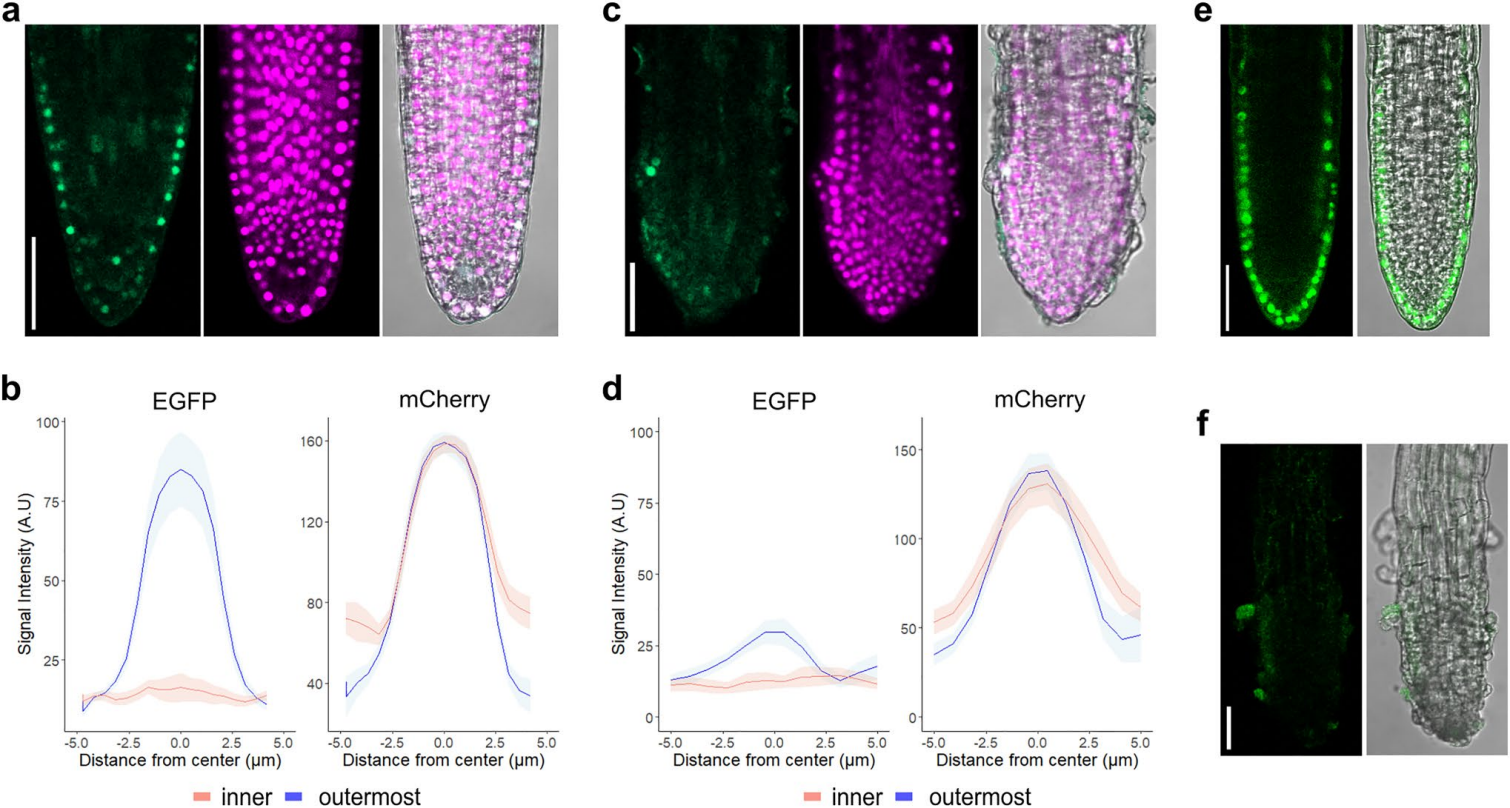
VLCFA-Cer molecules protect the PDF2 protein from degradation in the outermost LR cells. **a, c** Fluorescent images of EGFP (left) and mCherry (middle), and merged fluorescence and bright-field image (right) in LRs of *HSP::NLS-mCherry; HSP::PDF2-EGFP* plants 3 h post heat-pulse treatment. **b, d** Plots of EGFP (left) and mCherry (right) intensity in inner cells (*n* = 12) and outermost cells (*n* = 12). Seedlings without 0.3 µM cafenstrole treatment (mock treatment) are shown in (**a**) and (**b**); seedlings with 0.3 µM cafenstrole treatment are shown in (**c**) and (**d**). Mean data shown (mean ± s.e.m). **e, f** Fluorescent images of EGFP (left) and merged fluorescence and bright-field image (right) in LRs of *gPDF2-EGFP; atml1-3; pdf2-1* seedlings. Seedlings without 0.3 µM cafenstrole treatment (mock treatment) are shown in (**e**); seedlings treated with 0.3 µM cafenstrole are shown in (**f**). Scale bars: 50 μm

In our previous study, we identified a tryptophan residue located in the fourth alpha-helix of the START domain as a highly conserved amino acid residue among HD-ZIP IV TFs (Nagata et al. 2021). Based on amino acid alignments with the known ligand contact site of mammalian START domains, the conserved tryptophan is predicted to be required for the interaction between HD-ZIP IV TFs and their lipid-ligands (Nagata et al. 2021). A W-L substitution in the conserved tryptophan of ATML1 (W471L mutation; Fig. 1a, d) consistently attenuates the interaction between ATML1 and its lipid ligand VLCFA-Cer (Nagata et al. 2021). Thus, to examine the functional significance of the interaction between PDF2 and its lipid ligand VLCFA-Cer in the position-dependent regulation of PDF2 turnover, we introduced a W-L substitution into the conserved tryptophan residue of PDF2 (W463L mutation; Fig. 1a, d) and generated transgenic plants harboring the heat-inducible system of PDF2^W463L^-EGFP and NLS-mCherry (*HSP::NLS-mCherry; HSP::PDF2*^*W463L*^*-EGFP*). The heat-pulse treatment-dependent transcription of both transgenes was confirmed by RT-PCR (Fig. 1c). At 3 h post heat-pulse treatment, EGFP fluorescence was not detected either in the outermost LR cells or in the inner LR cells. However, mCherry fluorescence was still detected in all LR cells (Fig. 1g, h). These results suggest that the interaction between PDF2 and VLCFA-Cer is required for inhibition of the rapid turnover of PDF2 in the outermost LR cells. To confirm this hypothesis, we tested the effect of cafenstrole, a specific inhibitor of VLCFA biosynthesis (Nobusawa and Umeda 2012), on position-dependent regulation of PDF2 turnover. Cafenstrole treatment did not affect the activity of the *HSP* promoter (Fig. 1c). In the outermost LR cells of *HSP::NLS-mCherry; HSP::PDF2-EGFP* seedlings, the attenuation of EGFP fluorescence was significantly promoted by cafenstrole treatment (Fig. 2a–d), indicating that the depletion of VLCFA-Cers facilitates PDF2 turnover in the outermost LR cells as well as in the inner LR cells. Furthermore, cafenstrole treatment significantly reduced both the fluorescence intensity of EGFP and the number of cells expressing PDF2-EGFP in *gPDF2-EGFP; atml1-3; pdf2-1* seedlings (Fig. 2e, f). Collectively, these results demonstrate that the formation of the PDF2-VLCFA-Cer complex protects the PDF2 protein from degradation in the outermost LR cells.

**Fig. 3:**
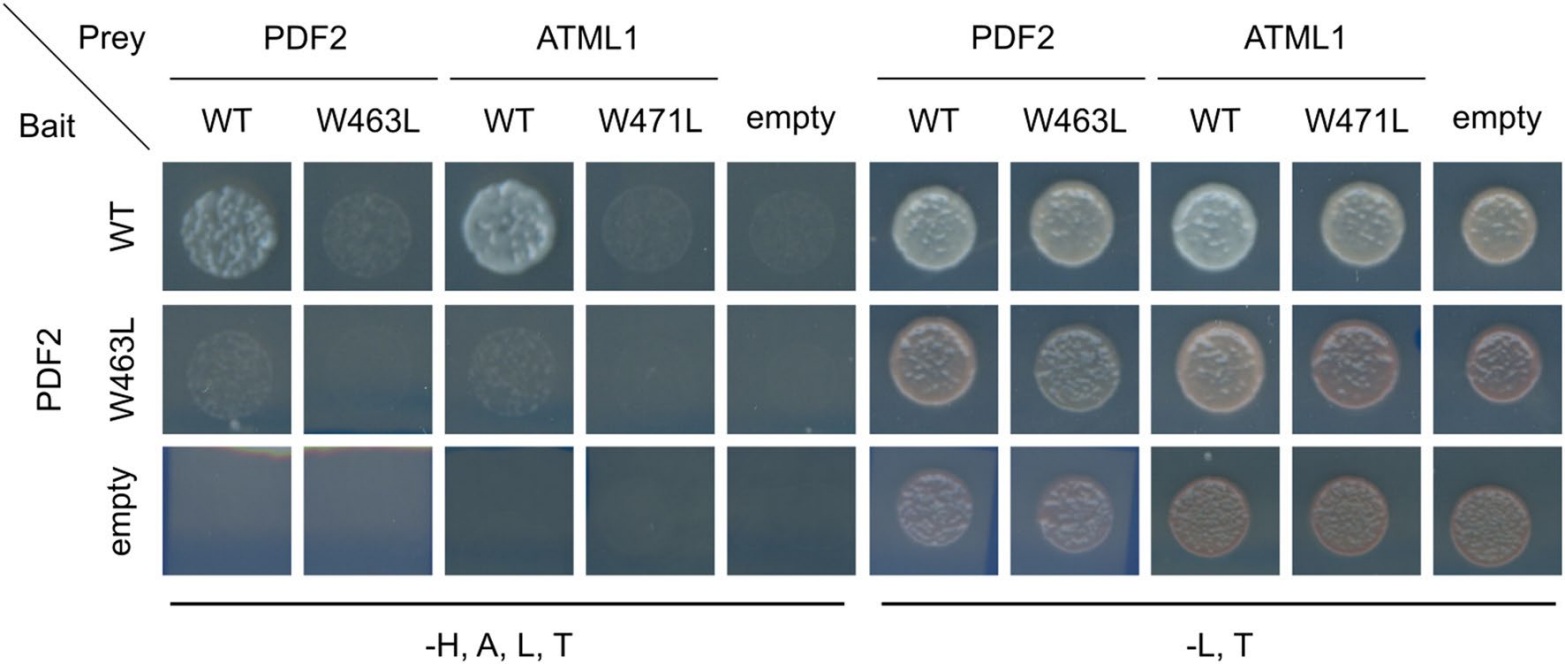
Lipid binding through the START domain affects the homo- and heterodimerization of PDF2 in yeast cells. Yeast two-hybrid assay of interaction among PDF2, PDF2^W463L^, ATML1, and ATML1^W471L^. Yeast cells were grown for 2 days on SD medium without Leu and Trp (-L, T); or without His, Ala, Leu, and Trp (-H, A, L, T)

HD-ZIP IV TFs, to which PDF2 and ATML1 belong, contain a ZIP motif that acts as a dimerization motif (Ariel et al. 2007). The homo- and heterodimerization of HD-ZIP IV TFs through the ZIP motif confer DNA-binding abilities and are thus required for their activities. The homodimerization of PDF2 and ATML1 and heterodimerization between PDF2 and ATML1 through their ZIP motifs have been shown in previous studies (Nagata and Abe 2021; Rombolá-Caldentey et al. 2014; San-Bento et al. 2014). Furthermore, our previous study demonstrated that the W471L mutation in ATML1 reduces the level of the ATML1 homodimer (Nagata and Abe 2021). Next, we examined whether the W463L mutation affects homodimerization of PDF2 and heterodimerization between PDF2 and ATML1 to further understand the functional significance of the interaction between PDF2 and VLCFA-Cer in the regulation of PDF2 activity. VLCFA-Cer molecules are an essential membrane component of yeast cells (Guillas et al. 2001). Thus, we employed a yeast two-hybrid system to understand the effect of PDF2-VLCFA-Cer complex formation on the dimerization of PDF2 (Chien et al. 1991). We found that yeast cells carrying BD-PDF2 with AD-PDF2 or AD-ATML1 were able to grow on the selection medium 2 days after incubation (Fig. 3). Conversely, yeast cells carrying AD-ATML1^W471L^ did not grow on the selection medium 2 days after incubation. Similarly, we found that yeast cells carrying BD-PDF2^W463L^ or AD-PDF2 ^W463L^ were unable to grow on the selection medium after 2 days of incubation (Fig. 3). Nevertheless, an additional incubation for 2 days resulted in the growth of yeast cells carrying BD-PDF2^W463L^ with AD-PDF2 or AD-ATML1 on the selection medium (Fig. S1). These results indicate that both the W463L mutation in PDF2 and the W471L mutation in ATML1 reduce the levels of PDF2 and ATML1 that exist in their homo- or heterodimeric forms in yeast cells. However, the W463L mutation in PDF2 does not completely eliminate the homo- or heterodimeric PDF2.

**Fig. 4:**
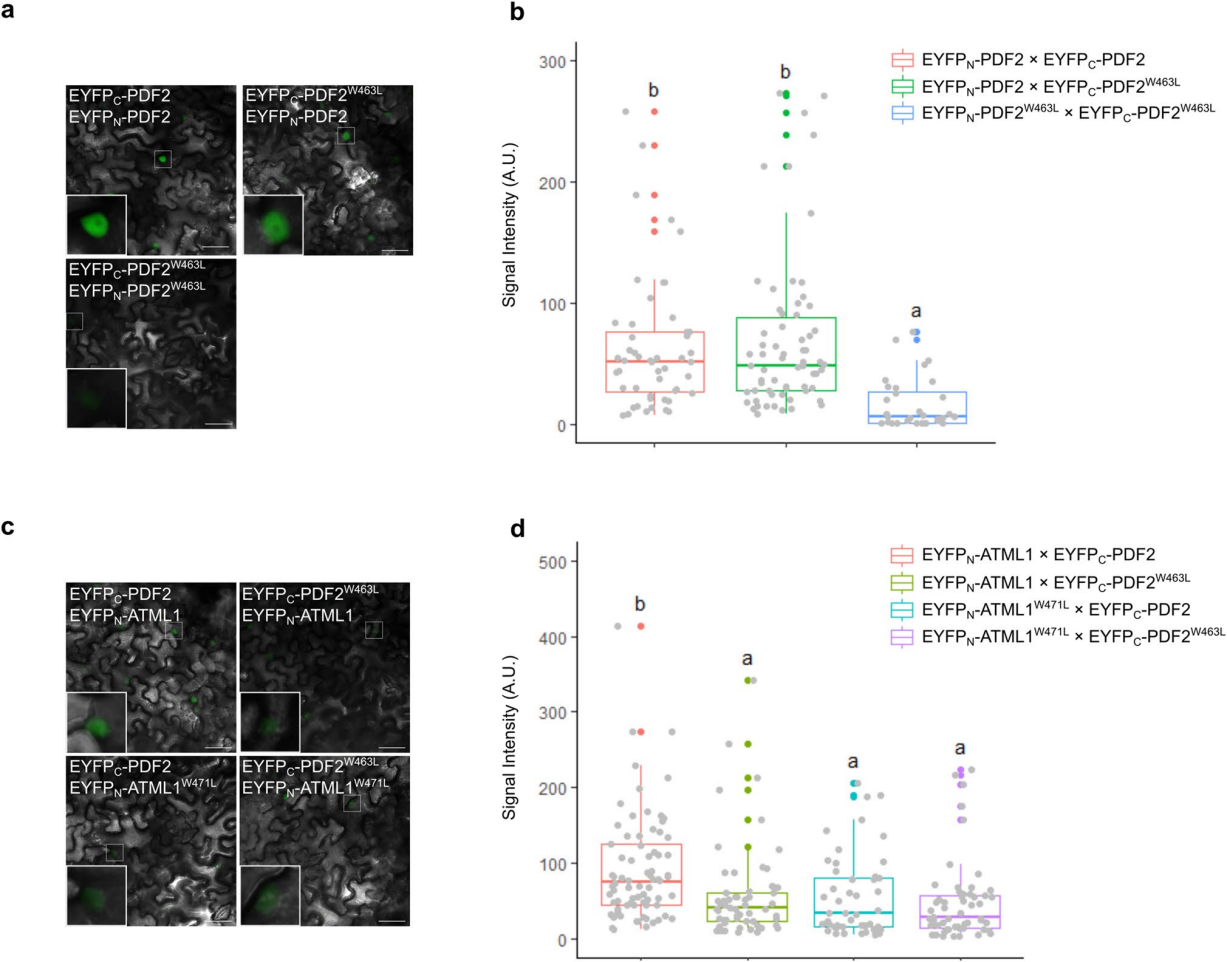
Lipid binding through the START domain affects the homo- and heterodimerization of PDF2 in plant cells. **a** BiFC assay of the interaction between PDF2 and PDF2^W463L^ in *Nicotiana benthamiana* leaves. The inset shows a magnified view of a representative region with BiFC signal (i.e., the region surrounded by the line). Scale bars, 50 µm. **b** Comparison of the BiFC signal intensity in (**a**), measured using ImageJ software. Different letters indicate statistically significant differences between means by the Tukey–Kramer's multiple comparison test (*p* < 0.05, *n* = 50, 66, and 32, respectively). **c** BiFC assay of the interaction among PDF2, PDF2^W463L^, ATML1, and ATML1^W471L^ in *Nicotiana benthamiana* leaves. The inset shows a magnified view of a representative region with BiFC signal (i.e., the region surrounded by the line). Scale bars, 50 µm. **d** Comparison of the BiFC signal intensity in (**c**), measured using ImageJ software. Different letters indicate statistically significant differences between means by the Tukey–Kramer’s multiple comparison test (*p* < 0.05, *n* = 76, 48, 57, and 56, respectively)

Next, we employed a transient expression system in *Nicotiana benthamiana* leaves to understand the effect of the PDF2-VLCFA-Cer complex formation on the dimerization of PDF2. Western blot analysis revealed that the accumulation of START domain mutants (PDF2^W463L^-EGFP and ATML1^W471L^-EGFP) was relatively lower than that of the wild-type proteins (PDF2-EGFP and ATML1-EGFP) when expressed under the control of the cauliflower mosaic virus 35S promoter in *Nicotiana benthamiana* leaves (Fig. S2). Thus, START domain mutants are relatively unstable in *Nicotiana benthamiana* leaves as well as in Arabidopsis LRs, indicating that *Nicotiana benthamiana* leaves provide a good system for understanding the behavior of PDF2 and ATML1 *in planta*. We then performed a bimolecular fluorescence complementation (BiFC) assay by agroinfiltration of *Nicotiana benthamiana* leaves. In this assay, ATML1 or PDF2 CDSs were translationally fused to the N-terminal half of enhanced yellow fluorescent protein (EYFP_N_) or the C-terminal half of EYFP (EYFP_C_). No fluorescent signal was observed when empty vectors were expressed in *Nicotiana benthamiana* leaves (Fig. S3). A strong fluorescent signal was observed in the epidermal nuclei of *Nicotiana benthamiana* co-expressing EYFP_C_-PDF2 with EYFP_N_-PDF2 or EYFP_N_-ATML1 (Fig. 4a, b), indicating that PDF2 dimerizes with PDF2 and ATML1 in plant cells. In contrast, the intensity of the fluorescent signal was significantly reduced in the nuclei of *Nicotiana benthamiana* co-expressing EYFP_N_-PDF2^W463L^ with EYFP_C_-PDF2^W463L^ (Fig. 4a, b). Similarly, the fluorescence signal intensity was significantly reduced in the nuclei of *Nicotiana benthamiana* expressing YFP_C_-PDF2^W463L^ or YFP_N_-ATML1^W471L^ (Fig. 4c, d). These results confirmed that the levels of the PDF2 homodimer and PDF2-ATML1 heterodimer were decreased by the inhibition of VLCFA-Cer binding to PDF2 and/or ATML1. Unexpectedly, the intensity of the fluorescent signal in the nuclei was not significantly changed in the nuclei of *Nicotiana benthamiana* co-expressing EYFP_N_-PDF2 with EYFP_C_-PDF2^W463L^ (Fig. 4a, b). We previously showed that the W471L mutation into ATML1 cause a decrease in the level of the ATML1 homodimer using the BiFC assay (Nagata and Abe 2021). Furthermore, the irreversibility of BiFC complexes has been well documented (Shyu and Hu 2008). Thus, a subtle decrease in the level of the PDF2 homodimer, caused by the W463L mutation, may not be detectable by the BiFC assay. Collectively, these results are consistent with our idea that the interaction between PDF2 and VLCFA-Cer is required for the maintenance of the level of the PDF2 dimer.

## Discussion

In this study, we examined the functional significance of the interaction between PDF2 and its lipid ligand VLCFA-Cer in the regulation of PDF2 activity. Previously, we reported that the interaction between ATML1 and VLCFA-Cer is required for the position-dependent regulation of ATML1 turnover and maintenance of the level of the ATML1 homodimer (Nagata and Abe 2021; Nagata et al. 2021). Consistently, our assay in which PDF2 was transiently induced by heat-pulse treatment revealed that the functional START domain of PDF2 and the biosynthesis of VLCFA-Cer in LR cells are both required for PDF2 stability in the outermost LR cells. Furthermore, Y2H and BiFC assays demonstrated that the functional START domain of PDF2 is required for maintaining the level of PDF2 that exists in the homo- or heterodimeric form. Thus, our analyses strongly support that the interactions between PDF2 and VLCFA-Cer and between ATML1 and VLCFA-Cer, are crucial for protein turnover and PDF2 dimer level maintenance, despite the specific role of PDF2 in the phase transition and specification of petal and stamen identities.

The molecular mechanism that regulates the turnover of PDF2 and ATML1 as well as affects the levels of dimeric PDF2 and ATML1, in a VLCFA-Cer-dependent manner, still needs to be addressed in future studies. In particular, it remains unclear whether the negative effects of the W463L mutation in PDF2 and the W471L mutation in ATML1 on the levels of dimeric PDF2 and ATML1 represent direct consequences of the compromised activity of their START domains. Our transient expression assay using *Nicotiana benthamiana* leaves established that protein accumulation was negatively affected by the introduction of the W463L mutation into PDF2 and the W471L mutation into ATML1. Thus, the negative effect of the W463L mutation in PDF2 and the W471L mutation in ATML1 on the levels of dimeric PDF2 and ATML1 may be attributed to the reduced accumulation of PDF2 and ATML1 in yeast and plant cells. The homodimerization of PHABULOSA (PHB), which belongs to the HD-ZIP III TF family, has been shown to be directly facilitated by its START domain activity (Husbands et al. 2022). Although extensive evolutionary divergence exists between HD-ZIP III and HD-ZIP IV TFs, it remains possible that the dimerization of PDF2 and ATML1 may be directly regulated by the activity of the START domain, as in PHB.

Altogether, our study supports the idea that a conserved VLCFA-Cer-mediated mechanism regulates the turnover of PDF2 and ATML1 and affects the levels of dimeric PDF2 and ATML1. Thus, the previously proposed model, in which the activities of PDF2 and ATML1 are spatially controlled by their lipid-ligand VLCFA-Cer during epidermal cell differentiation, is strongly underpinned. In addition, these results can explain the functional redundancy of PDF2 and ATML1 in epidermal cell differentiation during embryonic and vegetative development (Abe et al. 2001, 2003; Nagata et al. 2021; Ogawa et al. 2015).

However, whether PDF2 and ATML1 play completely redundant roles throughout the plant life cycle remains unknown. This is mainly due to a lack of knowledge about phenotypes, especially in the reproductive phase of the *atml1 pdf2* double mutant, which results in embryonic or seedling lethality (Abe et al. 2003; Ogawa et al. 2015). Given that the overexpression of PDF2 and ATML1 and mutations in *PDF2* and *ATML1* differently affect the flowering-related phenotypes, additional regulatory layers may alter the activity and/or function of PDF2 and ATML1 during reproductive development. Because our study demonstrates that the protein properties of PDF2 and ATML1 regulated by their lipid-ligand are highly conserved, it is plausible that the specific role of PDF2, if present, is attributed to the incorporation of a specific interactor of PDF2 into the dimeric HD-ZIP IV TF complex that contains PDF2. Indeed, previous studies have reported that several HD-ZIP IV TFs interact with specific proteins and the interactions are required for their specific functions (Horstman et al. 2015; Wu et al. 2011). Alternatively, the responsiveness to environmental and/or developmental cues at the transcriptional and/or translational level could be different between *PDF2* and *ATML1*. Considering that the VLCFA-Cer-mediated regulatory layer is involved in the robust patterning process that requires a precise positional signaling pathway, an unidentified regulatory layer may be involved in dynamic biological processes including response to fluctuating environmental stimuli.

## Supplementary Information

Below is the link to the electronic supplementary material.

Supplementary file1 (PDF 252 KB)

## Data Availability

The datasets generated during and/or analysed during the current study are available from the corresponding author on reasonable request.
