# Supplementary material for: A conserved mechanism determines the activity of two pivotal transcription factors that control epidermal cell differentiation in Arabidopsis thaliana
Source: J Plant Res. 2023 Feb 24;136(3):349–58. doi: 10.1007/s10265-023-01439-7 (PMC10126025; doi:10.1007/s10265-023-01439-7)
Supplement: Supplementary file 1 — Supplementary file1 (PDF 252 KB) [file 10265_2023_1439_MOESM1_ESM.pdf]

Supplementary Information (SI)

Journal name: Journal of Plant Research

Title: A conserved mechanism determines the activity of two pivotal transcription factors that control epidermal cell differentiation in *Arabidopsis thaliana*

Author names: Kenji Nagata, Mitsutomo Abe

correspondence: Mitsutomo Abe, Department of Life Sciences, Graduate School of Arts and Sciences, University of Tokyo, 3-8-1 Komaba, Meguro-ku, Tokyo 153-8902, Japan.

Email: mabe@bio.c.u-tokyo.ac.jp

This PDF file includes:

Table S1 and S2

Fig. S1–S3

Table S1: List of primers used for plasmid construction

Name	Sequence (5’ to 3’)
PDF2-m1-F	AGTTCTTCACTGTTGATACATATGTACCATCCAAACATGTT
PDF2-m1-R	TGTAGCCACCAAACGTTTCGCACCGAAAGCT
PDF2-m2-F	CGAAACGTTTGGTGGCTACACTCGAACGAC
PDF2-m2-R	GAGTTGTTGATTCAGAATTGCTACGCTCCTCCTCCAACAT

Table S2: List of primers and PCR conditions used for RT-PCR analysis

Gene	Forward primer (5' to 3')	Reverse primer (5' to 3')	annealing temp.	cycles
PDF2-EGFP	TATGTGATTTACGCGCCAGTG	CTCGCCCTTGCTCACCATAC	55	30
NLS-mCherry	GACCCCCAAAGAAGAAGCGTA	CTGCTTGATCTCGCCCTTCA	55	30
TUB2	CTCAAGAGGTTCTCAGCAGTA	TCACCTTCTTCATCCGCAGTT	55	30

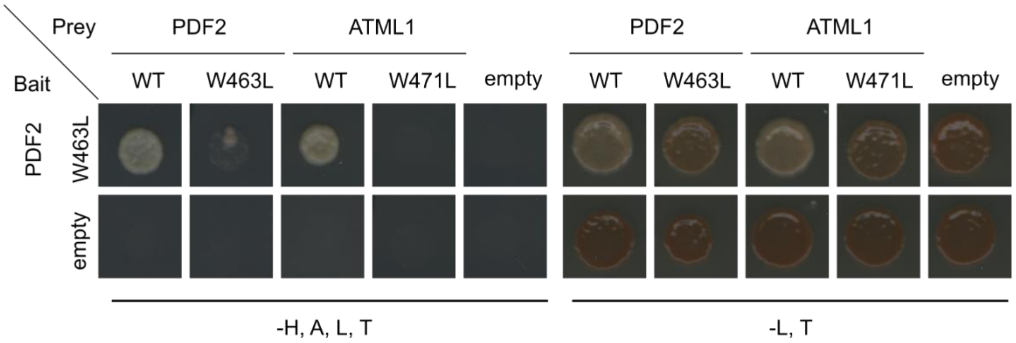

Fig. S1 Y2H assay of interaction among PDF2, PDF2^{W463L}, ATML1, and ATML1^{W471L}. Yeast cells were grown for four days on SD medium without Leu and Trp (-L, T) or without His, Ala, Leu, and Trp (-H, A, L, T).

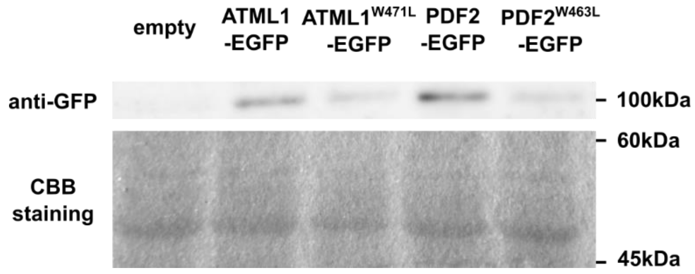

Fig. S2 Transient overexpression assay of ATML1-EGFP, ATML1^{W471L}-EGFP, PDF2-EGFP or PDF2^{W463L}-EGFP. ATML1-EGFP, ATML1^{W471L}-EGFP, PDF2-EGFP, and PDF2^{W463L}-EGFP were expressed in *Nicotiana benthamiana* leaves, and the protein levels were monitored by western blotting. Coomassie Brilliant Blue-stained membranes were used as loading controls.

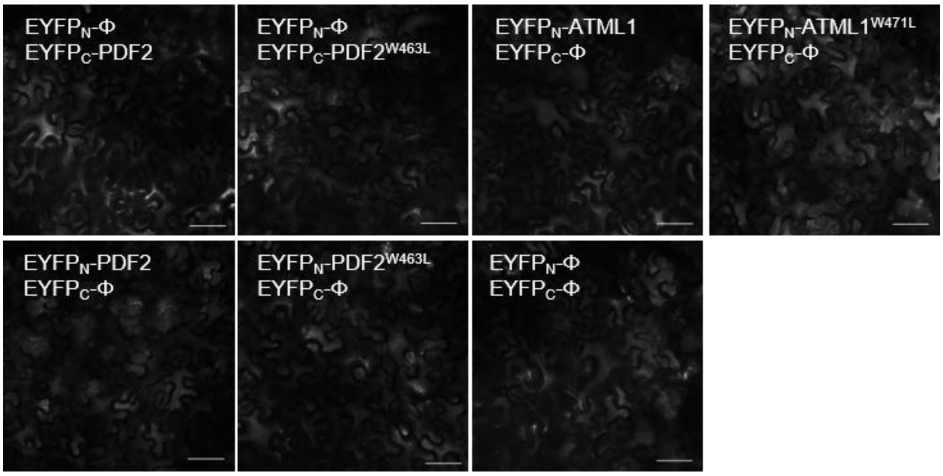

Fig. S3 BiFC negative assays. EYFP$_N$-Ø and EYFP$_C$-Ø indicate empty vectors (i.e., p35S::EYFP$_N$ or p35S::EYEP$_C$ (Nagata and Abe. 2021) vector, respectively). Scale bars: 50 µm.
